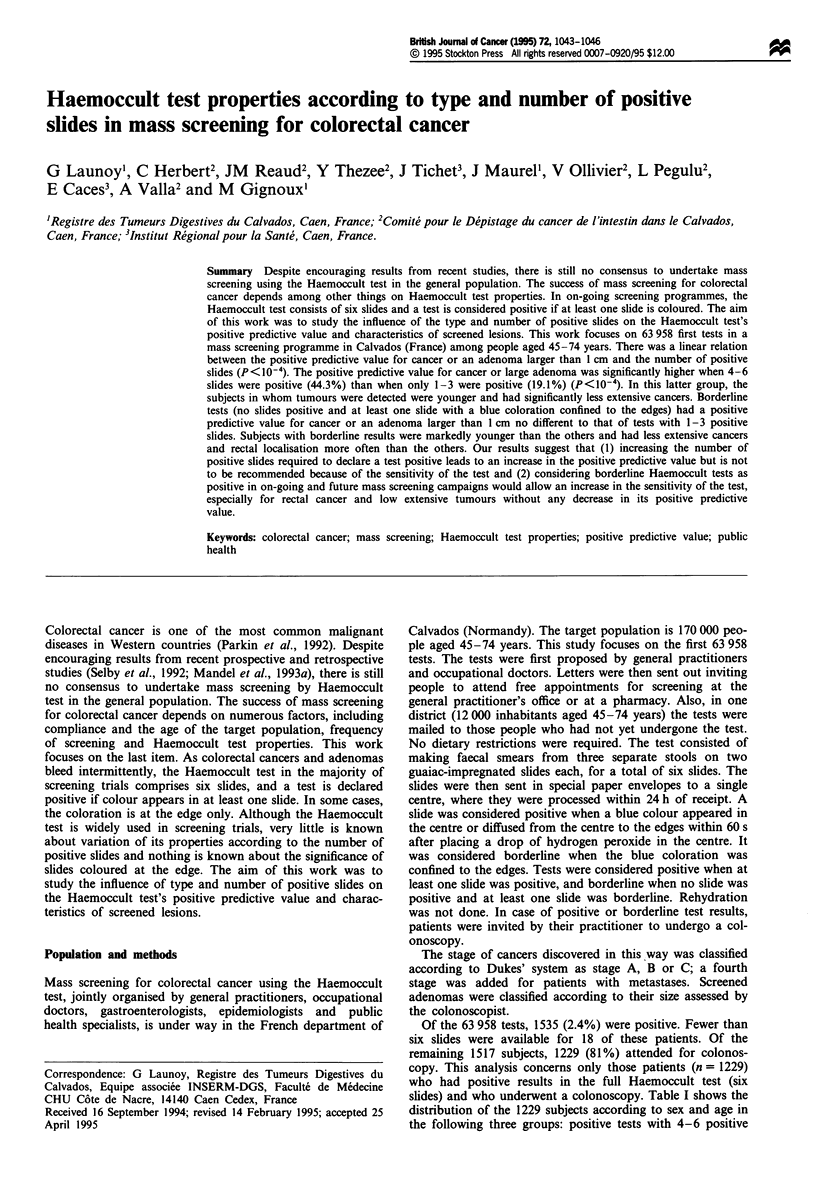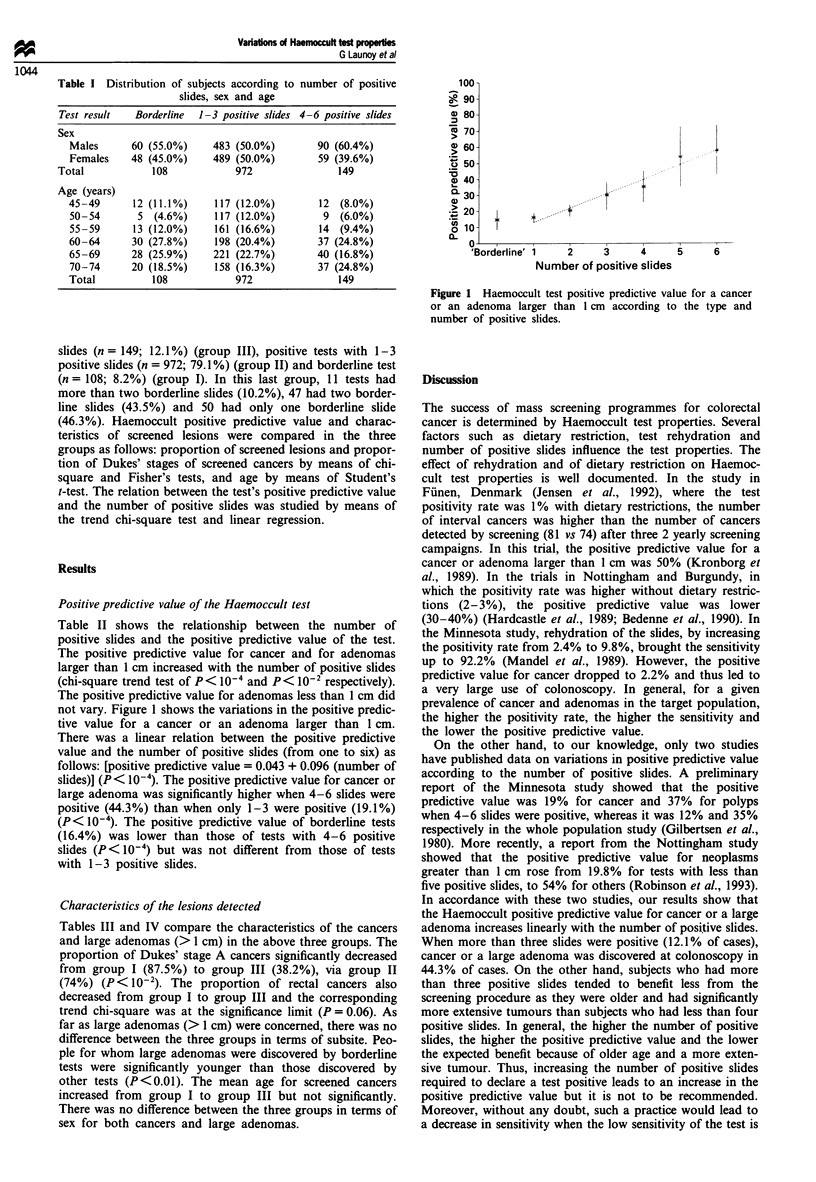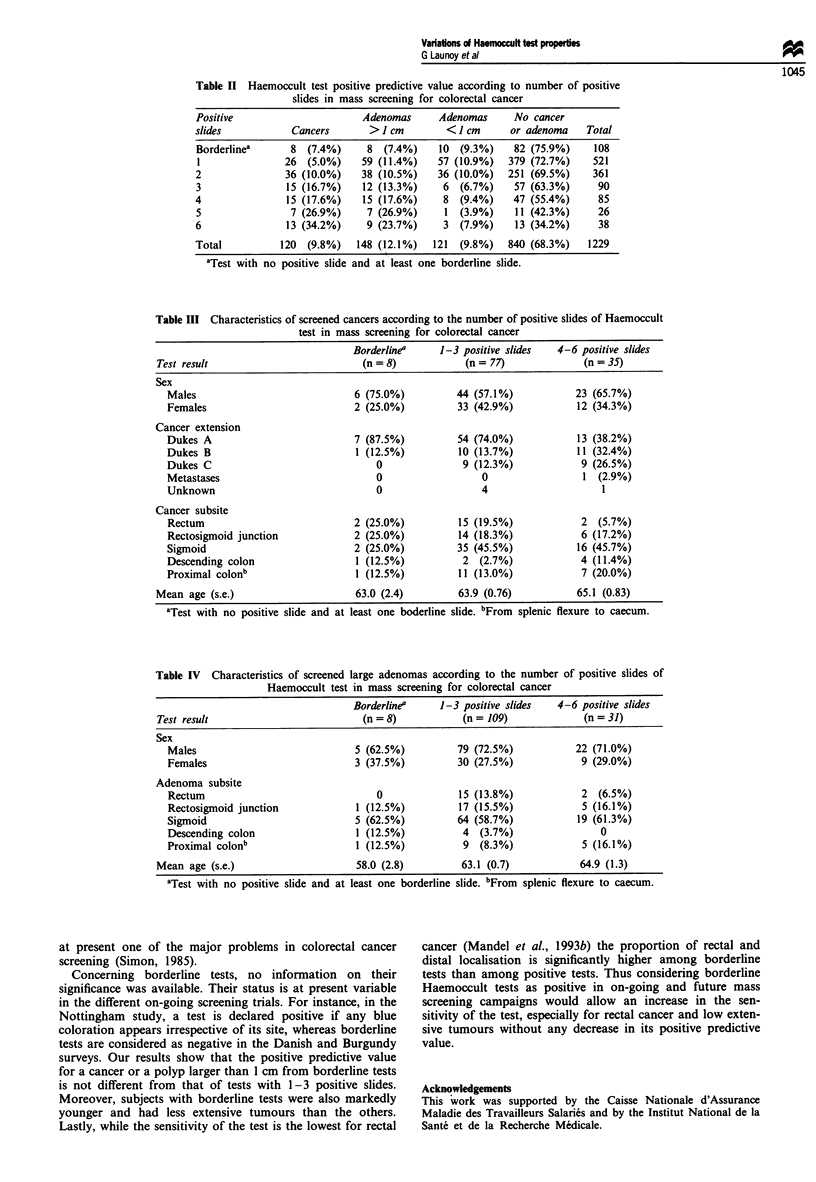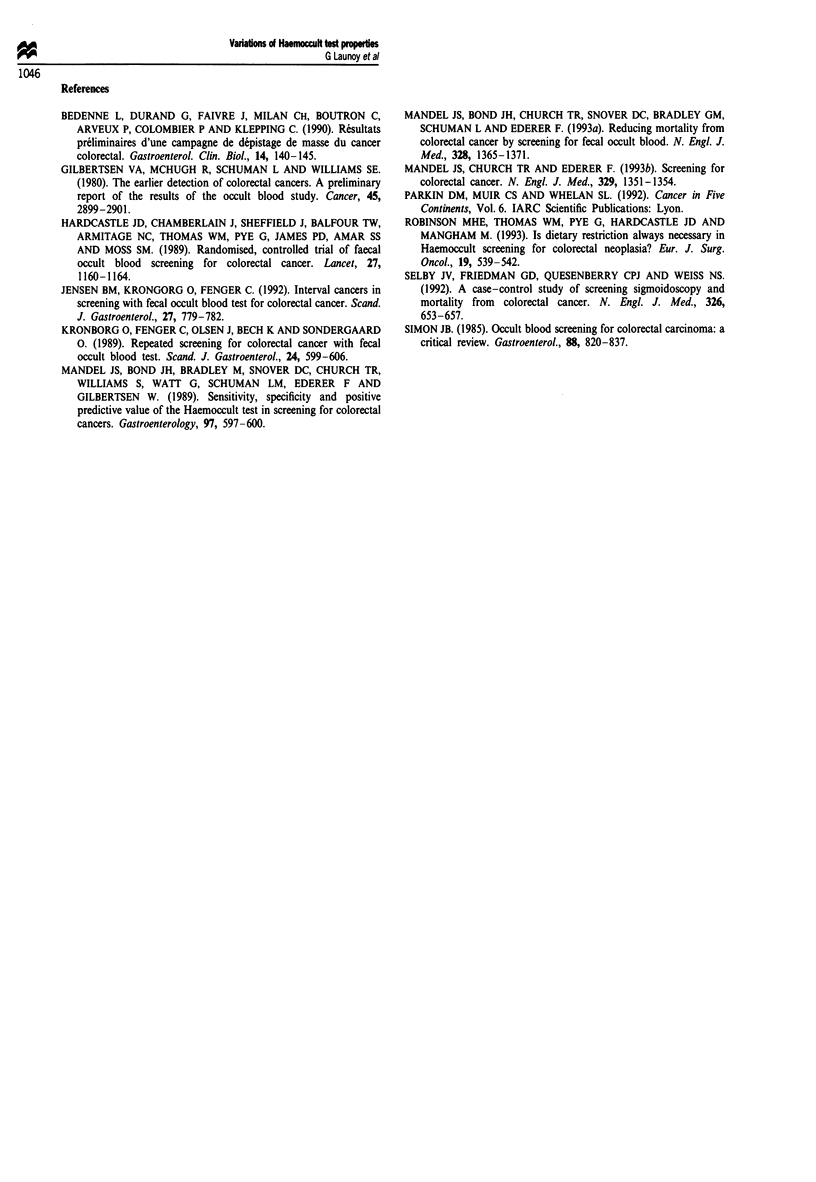# Haemoccult test properties according to type and number of positive slides in mass screening for colorectal cancer.

**DOI:** 10.1038/bjc.1995.459

**Published:** 1995-10

**Authors:** G. Launoy, C. Herbert, J. M. Reaud, Y. Thezee, J. Tichet, J. Maurel, V. Ollivier, L. Pegulu, E. Caces, A. Valla

**Affiliations:** Registre des Tumeurs Digestives du Calvados, Caen, France.

## Abstract

Despite encouraging results from recent studies, there is still no consensus to undertake mass screening using the Haemoccult test in the general population. The success of mass screening for colorectal cancer depends among other things on Haemoccult test properties. In on-going screening programmes, the Haemoccult test consists of six slides and a test is considered positive if at least one slide is coloured. The aim of this work was to study the influence of the type and number of positive slides on the Haemoccult test's positive predictive value and characteristics of screened lesions. This work focuses on 63,958 first tests in a mass screening programme in Calvados (France) among people aged 45-74 years. There was a linear relation between the positive predictive value for cancer or an adenoma larger than 1 cm and the number of positive slides (P < 10(-4)). The positive predictive value for cancer or large adenoma was significantly higher when 4-6 slides were positive (44.3%) than when only 1-3 were positive (19.1%) (P < 10(-4)). In this latter group, the subjects in whom tumours were detected were younger and had significantly less extensive cancers. Borderline tests (no slides positive and at least one slide with a blue coloration confined to the edges) had a positive predictive value for cancer or an adenoma larger than 1 cm no different to that of tests with 1-3 positive slides. Subjects with borderline results were markedly younger than the others and had less extensive cancers and rectal localisation more often than the others. Our results suggest that (1) increasing the number of positive slides required to declare a test positive leads to an increase in the positive predictive value but is not to be recommended because of the sensitivity of the test and (2) considering borderline Haemoccult tests as positive in on-going and future mass screening campaigns would allow an increase in the sensitivity of the test, especially for rectal cancer and low extensive tumours without any decrease in its positive predictive value.


British Journal of Cancer (1995) 72, 1043–1046
© 1995 Stockton Press All rights reserved 0007-0920/95 $12.00

### Haemoccult test properties according to type and number of positive slides in mass screening for colorectal cancer

G Launoy[1], C Herbert[2], JM Reaud[2], Y Thezee[2], J Tichet[3], J Maurel[1], V Ollivier[2], L Pegulu[2], E Caces[3], A Valla[2] and M Gignoux[1]

*[1]Registre des Tumeurs Digestives du Calvados, Caen, France; [2]Comité pour le Dépistage du cancer de l'intestin dans le Calvados, Caen, France; [3]Institut Régional pour la Santé, Caen, France.*

**Summary** Despite encouraging results from recent studies, there is still no consensus to undertake mass screening using the Haemoccult test in the general population. The success of mass screening for colorectal cancer depends among other things on Haemoccult test properties. In on-going screening programmes, the Haemoccult test consists of six slides and a test is considered positive if at least one slide is coloured. The aim of this work was to study the influence of the type and number of positive slides on the Haemoccult test's positive predictive value and characteristics of screened lesions. This work focuses on 63 958 first tests in a mass screening programme in Calvados (France) among people aged 45–74 years. There was a linear relation between the positive predictive value for cancer or an adenoma larger than 1 cm and the number of positive slides ($P<10^{-4}$). The positive predictive value for cancer or large adenoma was significantly higher when 4–6 slides were positive (44.3%) than when only 1–3 were positive (19.1%) ($P<10^{-4}$). In this latter group, the subjects in whom tumours were detected were younger and had significantly less extensive cancers. Borderline tests (no slides positive and at least one slide with a blue coloration confined to the edges) had a positive predictive value for cancer or an adenoma larger than 1 cm no different to that of tests with 1–3 positive slides. Subjects with borderline results were markedly younger than the others and had less extensive cancers and rectal localisation more often than the others. Our results suggest that (1) increasing the number of positive slides required to declare a test positive leads to an increase in the positive predictive value but is not to be recommended because of the sensitivity of the test and (2) considering borderline Haemoccult tests as positive in on-going and future mass screening campaigns would allow an increase in the sensitivity of the test, especially for rectal cancer and low extensive tumours without any decrease in its positive predictive value.

**Keywords:** colorectal cancer; mass screening; Haemoccult test properties; positive predictive value; public health

Colorectal cancer is one of the most common malignant diseases in Western countries (Parkin *et al.*, 1992). Despite encouraging results from recent prospective and retrospective studies (Selby *et al.*, 1992; Mandel *et al.*, 1993*a*), there is still no consensus to undertake mass screening by Haemoccult test in the general population. The success of mass screening for colorectal cancer depends on numerous factors, including compliance and the age of the target population, frequency of screening and Haemoccult test properties. This work focuses on the last item. As colorectal cancers and adenomas bleed intermittently, the Haemoccult test in the majority of screening trials comprises six slides, and a test is declared positive if colour appears in at least one slide. In some cases, the coloration is at the edge only. Although the Haemoccult test is widely used in screening trials, very little is known about variation of its properties according to the number of positive slides and nothing is known about the significance of slides coloured at the edge. The aim of this work was to study the influence of type and number of positive slides on the Haemoccult test's positive predictive value and characteristics of screened lesions.

### Population and methods

Mass screening for colorectal cancer using the Haemoccult test, jointly organised by general practitioners, occupational doctors, gastroenterologists, epidemiologists and public health specialists, is under way in the French department of Calvados (Normandy). The target population is 170 000 people aged 45–74 years. This study focuses on the first 63 958 tests. The tests were first proposed by general practitioners and occupational doctors. Letters were then sent out inviting people to attend free appointments for screening at the general practitioner's office or at a pharmacy. Also, in one district (12 000 inhabitants aged 45–74 years) the tests were mailed to those people who had not yet undergone the test. No dietary restrictions were required. The test consisted of making faecal smears from three separate stools on two guaiac-impregnated slides each, for a total of six slides. The slides were then sent in special paper envelopes to a single centre, where they were processed within 24 h of receipt. A slide was considered positive when a blue colour appeared in the centre or diffused from the centre to the edges within 60 s after placing a drop of hydrogen peroxide in the centre. It was considered borderline when the blue coloration was confined to the edges. Tests were considered positive when at least one slide was positive, and borderline when no slide was positive and at least one slide was borderline. Rehydration was not done. In case of positive or borderline test results, patients were invited by their practitioner to undergo a colonoscopy.

The stage of cancers discovered in this way was classified according to Dukes' system as stage A, B or C; a fourth stage was added for patients with metastases. Screened adenomas were classified according to their size assessed by the colonoscopist.

Of the 63 958 tests, 1535 (2.4%) were positive. Fewer than six slides were available for 18 of these patients. Of the remaining 1517 subjects, 1229 (81%) attended for colonoscopy. This analysis concerns only those patients ($n = 1229$) who had positive results in the full Haemoccult test (six slides) and who underwent a colonoscopy. Table I shows the distribution of the 1229 subjects according to sex and age in the following three groups: positive tests with 4–6 positive

Correspondence: G Launoy, Registre des Tumeurs Digestives du Calvados, Equipe associée INSERM-DGS, Faculté de Médecine CHU Côte de Nacre, 14140 Caen Cedex, France
Received 16 September 1994; revised 14 February 1995; accepted 25 April 1995

Table I Distribution of subjects according to number of positive slides, sex and age

| Test result | Borderline | 1-3 positive slides | 4-6 positive slides |
|---|---|---|---|
| Sex | | | |
| Males | 60 (55.0%) | 483 (50.0%) | 90 (60.4%) |
| Females | 48 (45.0%) | 489 (50.0%) | 59 (39.6%) |
| Total | 108 | 972 | 149 |
| Age (years) | | | |
| 45-49 | 12 (11.1%) | 117 (12.0%) | 12 (8.0%) |
| 50-54 | 5 (4.6%) | 117 (12.0%) | 9 (6.0%) |
| 55-59 | 13 (12.0%) | 161 (16.6%) | 14 (9.4%) |
| 60-64 | 30 (27.8%) | 198 (20.4%) | 37 (24.8%) |
| 65-69 | 28 (25.9%) | 221 (22.7%) | 40 (16.8%) |
| 70-74 | 20 (18.5%) | 158 (16.3%) | 37 (24.8%) |
| Total | 108 | 972 | 149 |

**Figure 1** Haemoccult test positive predictive value for a cancer or an adenoma larger than 1 cm according to the type and number of positive slides.

slides ($n = 149$; 12.1%) (group III), positive tests with 1-3 positive slides ($n = 972$; 79.1%) (group II) and borderline test ($n = 108$; 8.2%) (group I). In this last group, 11 tests had more than two borderline slides (10.2%), 47 had two borderline slides (43.5%) and 50 had only one borderline slide (46.3%). Haemoccult positive predictive value and characteristics of screened lesions were compared in the three groups as follows: proportion of screened lesions and proportion of Dukes' stages of screened cancers by means of chi-square and Fisher's tests, and age by means of Student's $t$-test. The relation between the test's positive predictive value and the number of positive slides was studied by means of the trend chi-square test and linear regression.

### Results

### *Positive predictive value of the Haemoccult test*

Table II shows the relationship between the number of positive slides and the positive predictive value of the test. The positive predictive value for cancer and for adenomas larger than 1 cm increased with the number of positive slides (chi-square trend test of $P < 10^{-4}$ and $P < 10^{-2}$ respectively). The positive predictive value for adenomas less than 1 cm did not vary. Figure 1 shows the variations in the positive predictive value for a cancer or an adenoma larger than 1 cm. There was a linear relation between the positive predictive value and the number of positive slides (from one to six) as follows: [positive predictive value = 0.043 + 0.096 (number of slides)] ($P < 10^{-4}$). The positive predictive value for cancer or large adenoma was significantly higher when 4-6 slides were positive (44.3%) than when only 1-3 were positive (19.1%) ($P < 10^{-4}$). The positive predictive value of borderline tests (16.4%) was lower than those of tests with 4-6 positive slides ($P < 10^{-4}$) but was not different from those of tests with 1-3 positive slides.

### *Characteristics of the lesions detected*

Tables III and IV compare the characteristics of the cancers and large adenomas (> 1 cm) in the above three groups. The proportion of Dukes' stage A cancers significantly decreased from group I (87.5%) to group III (38.2%), via group II (74%) ($P < 10^{-2}$). The proportion of rectal cancers also decreased from group I to group III and the corresponding trend chi-square was at the significance limit ($P = 0.06$). As far as large adenomas (> 1 cm) were concerned, there was no difference between the three groups in terms of subsite. People for whom large adenomas were discovered by borderline tests were significantly younger than those discovered by other tests ($P < 0.01$). The mean age for screened cancers increased from group I to group III but not significantly. There was no difference between the three groups in terms of sex for both cancers and large adenomas.

### Discussion

The success of mass screening programmes for colorectal cancer is determined by Haemoccult test properties. Several factors such as dietary restriction, test rehydration and number of positive slides influence the test properties. The effect of rehydration and of dietary restriction on Haemoccult test properties is well documented. In the study in Fünen, Denmark (Jensen *et al.*, 1992), where the test positivity rate was 1% with dietary restrictions, the number of interval cancers was higher than the number of cancers detected by screening (81 *vs* 74) after three 2 yearly screening campaigns. In this trial, the positive predictive value for a cancer or adenoma larger than 1 cm was 50% (Kronborg *et al.*, 1989). In the trials in Nottingham and Burgundy, in which the positivity rate was higher without dietary restrictions (2-3%), the positive predictive value was lower (30-40%) (Hardcastle *et al.*, 1989; Bedenne *et al.*, 1990). In the Minnesota study, rehydration of the slides, by increasing the positivity rate from 2.4% to 9.8%, brought the sensitivity up to 92.2% (Mandel *et al.*, 1989). However, the positive predictive value for cancer dropped to 2.2% and thus led to a very large use of colonoscopy. In general, for a given prevalence of cancer and adenomas in the target population, the higher the positivity rate, the higher the sensitivity and the lower the positive predictive value.

On the other hand, to our knowledge, only two studies have published data on variations in positive predictive value according to the number of positive slides. A preliminary report of the Minnesota study showed that the positive predictive value was 19% for cancer and 37% for polyps when 4-6 slides were positive, whereas it was 12% and 35% respectively in the whole population study (Gilbertsen *et al.*, 1980). More recently, a report from the Nottingham study showed that the positive predictive value for neoplasms greater than 1 cm rose from 19.8% for tests with less than five positive slides, to 54% for others (Robinson *et al.*, 1993). In accordance with these two studies, our results show that the Haemoccult positive predictive value for cancer or a large adenoma increases linearly with the number of positive slides. When more than three slides were positive (12.1% of cases), cancer or a large adenoma was discovered at colonoscopy in 44.3% of cases. On the other hand, subjects who had more than three positive slides tended to benefit less from the screening procedure as they were older and had significantly more extensive tumours than subjects who had less than four positive slides. In general, the higher the number of positive slides, the higher the positive predictive value and the lower the expected benefit because of older age and a more extensive tumour. Thus, increasing the number of positive slides required to declare a test positive leads to an increase in the positive predictive value but it is not to be recommended. Moreover, without any doubt, such a practice would lead to a decrease in sensitivity when the low sensitivity of the test is

**Table II** Haemoccult test positive predictive value according to number of positive slides in mass screening for colorectal cancer

| *Positive slides* | *Cancers* | *Adenomas >1 cm* | *Adenomas <1 cm* | *No cancer or adenoma* | *Total* |
|---|---|---|---|---|---|
| Borderline[a] | 8 (7.4%) | 8 (7.4%) | 10 (9.3%) | 82 (75.9%) | 108 |
| 1 | 26 (5.0%) | 59 (11.4%) | 57 (10.9%) | 379 (72.7%) | 521 |
| 2 | 36 (10.0%) | 38 (10.5%) | 36 (10.0%) | 251 (69.5%) | 361 |
| 3 | 15 (16.7%) | 12 (13.3%) | 6 (6.7%) | 57 (63.3%) | 90 |
| 4 | 15 (17.6%) | 15 (17.6%) | 8 (9.4%) | 47 (55.4%) | 85 |
| 5 | 7 (26.9%) | 7 (26.9%) | 1 (3.9%) | 11 (42.3%) | 26 |
| 6 | 13 (34.2%) | 9 (23.7%) | 3 (7.9%) | 13 (34.2%) | 38 |
| Total | 120 (9.8%) | 148 (12.1%) | 121 (9.8%) | 840 (68.3%) | 1229 |

[a]Test with no positive slide and at least one borderline slide.

**Table III** Characteristics of screened cancers according to the number of positive slides of Haemoccult test in mass screening for colorectal cancer

| *Test result* | *Borderline*[a] (n = *8*) | *1–3 positive slides* (n = *77*) | *4–6 positive slides* (n = *35*) |
|---|---|---|---|
| Sex | | | |
| Males | 6 (75.0%) | 44 (57.1%) | 23 (65.7%) |
| Females | 2 (25.0%) | 33 (42.9%) | 12 (34.3%) |
| Cancer extension | | | |
| Dukes A | 7 (87.5%) | 54 (74.0%) | 13 (38.2%) |
| Dukes B | 1 (12.5%) | 10 (13.7%) | 11 (32.4%) |
| Dukes C | 0 | 9 (12.3%) | 9 (26.5%) |
| Metastases | 0 | 0 | 1 (2.9%) |
| Unknown | 0 | 4 | 1 |
| Cancer subsite | | | |
| Rectum | 2 (25.0%) | 15 (19.5%) | 2 (5.7%) |
| Rectosigmoid junction | 2 (25.0%) | 14 (18.3%) | 6 (17.2%) |
| Sigmoid | 2 (25.0%) | 35 (45.5%) | 16 (45.7%) |
| Descending colon | 1 (12.5%) | 2 (2.7%) | 4 (11.4%) |
| Proximal colon[b] | 1 (12.5%) | 11 (13.0%) | 7 (20.0%) |
| Mean age (s.e.) | 63.0 (2.4) | 63.9 (0.76) | 65.1 (0.83) |

[a]Test with no positive slide and at least one boderline slide. [b]From splenic flexure to caecum.

**Table IV** Characteristics of screened large adenomas according to the number of positive slides of Haemoccult test in mass screening for colorectal cancer

| *Test result* | *Borderline*[a] (n = *8*) | *1–3 positive slides* (n = *109*) | *4–6 positive slides* (n = *31*) |
|---|---|---|---|
| Sex | | | |
| Males | 5 (62.5%) | 79 (72.5%) | 22 (71.0%) |
| Females | 3 (37.5%) | 30 (27.5%) | 9 (29.0%) |
| Adenoma subsite | | | |
| Rectum | 0 | 15 (13.8%) | 2 (6.5%) |
| Rectosigmoid junction | 1 (12.5%) | 17 (15.5%) | 5 (16.1%) |
| Sigmoid | 5 (62.5%) | 64 (58.7%) | 19 (61.3%) |
| Descending colon | 1 (12.5%) | 4 (3.7%) | 0 |
| Proximal colon[b] | 1 (12.5%) | 9 (8.3%) | 5 (16.1%) |
| Mean age (s.e.) | 58.0 (2.8) | 63.1 (0.7) | 64.9 (1.3) |

[a]Test with no positive slide and at least one borderline slide. [b]From splenic flexure to caecum.

at present one of the major problems in colorectal cancer screening (Simon, 1985).

Concerning borderline tests, no information on their significance was available. Their status is at present variable in the different on-going screening trials. For instance, in the Nottingham study, a test is declared positive if any blue coloration appears irrespective of its site, whereas borderline tests are considered as negative in the Danish and Burgundy surveys. Our results show that the positive predictive value for a cancer or a polyp larger than 1 cm from borderline tests is not different from that of tests with 1–3 positive slides. Moreover, subjects with borderline tests were also markedly younger and had less extensive tumours than the others. Lastly, while the sensitivity of the test is the lowest for rectal cancer (Mandel *et al.*, 1993*b*) the proportion of rectal and distal localisation is significantly higher among borderline tests than among positive tests. Thus considering borderline Haemoccult tests as positive in on-going and future mass screening campaigns would allow an increase in the sensitivity of the test, especially for rectal cancer and low extensive tumours without any decrease in its positive predictive value.

**Acknowledgements**

This work was supported by the Caisse Nationale d'Assurance Maladie des Travailleurs Salariés and by the Institut National de la Santé et de la Recherche Médicale.

### References

BEDENNE L, DURAND G, FAIVRE J, MILAN CH, BOUTRON C, ARVEUX P, COLOMBIER P AND KLEPPING C. (1990). Résultats préliminaires d'une campagne de dépistage de masse du cancer colorectal. *Gastroenterol. Clin. Biol.*, **14**, 140–145.

GILBERTSEN VA, MCHUGH R, SCHUMAN L AND WILLIAMS SE. (1980). The earlier detection of colorectal cancers. A preliminary report of the results of the occult blood study. *Cancer*, **45**, 2899–2901.

HARDCASTLE JD, CHAMBERLAIN J, SHEFFIELD J, BALFOUR TW, ARMITAGE NC, THOMAS WM, PYE G, JAMES PD, AMAR SS AND MOSS SM. (1989). Randomised, controlled trial of faecal occult blood screening for colorectal cancer. *Lancet*, **27**, 1160–1164.

JENSEN BM, KRONGORG O, FENGER C. (1992). Interval cancers in screening with fecal occult blood test for colorectal cancer. *Scand. J. Gastroenterol.*, **27**, 779–782.

KRONBORG O, FENGER C, OLSEN J, BECH K AND SONDERGAARD O. (1989). Repeated screening for colorectal cancer with fecal occult blood test. *Scand. J. Gastroenterol.*, **24**, 599–606.

MANDEL JS, BOND JH, BRADLEY M, SNOVER DC, CHURCH TR, WILLIAMS S, WATT G, SCHUMAN LM, EDERER F AND GILBERTSEN W. (1989). Sensitivity, specificity and positive predictive value of the Haemoccult test in screening for colorectal cancers. *Gastroenterology*, **97**, 597–600.

MANDEL JS, BOND JH, CHURCH TR, SNOVER DC, BRADLEY GM, SCHUMAN L AND EDERER F. (1993*a*). Reducing mortality from colorectal cancer by screening for fecal occult blood. *N. Engl. J. Med.*, **328**, 1365–1371.

MANDEL JS, CHURCH TR AND EDERER F. (1993*b*). Screening for colorectal cancer. *N. Engl. J. Med.*, **329**, 1351–1354.

PARKIN DM, MUIR CS AND WHELAN SL. (1992). *Cancer in Five Continents*, Vol. 6. IARC Scientific Publications: Lyon.

ROBINSON MHE, THOMAS WM, PYE G, HARDCASTLE JD AND MANGHAM M. (1993). Is dietary restriction always necessary in Haemoccult screening for colorectal neoplasia? *Eur. J. Surg. Oncol.*, **19**, 539–542.

SELBY JV, FRIEDMAN GD, QUESENBERRY CPJ AND WEISS NS. (1992). A case-control study of screening sigmoidoscopy and mortality from colorectal cancer. *N. Engl. J. Med.*, **326**, 653–657.

SIMON JB. (1985). Occult blood screening for colorectal carcinoma: a critical review. *Gastroenterol.*, **88**, 820–837.